# Scope, quality and inclusivity of international clinical guidelines on mental health and substance abuse in relation to dual diagnosis, social and community outcomes: a systematic review

**DOI:** 10.1186/s12888-021-03188-0

**Published:** 2021-04-23

**Authors:** Ray Alsuhaibani, Douglas Cary Smith, Richard Lowrie, Sumayah Aljhani, Vibhu Paudyal

**Affiliations:** 1 School of Biosciences, University of Birmingham, Edgbaston, Birmingham, B15 2TT UK; 2 Department of Pharmacology and Toxicology, College of Pharmacy, Qassim University, 51 452 Qassim, Kingdom of Saudi Arabia; 3 School of Social Work, University of Illinois at Urbana-Champaign, Champaign, IL USA; 4 Homeless Health, Pharmacy Services, NHS Greater Glasgow and Clyde, Glasgow, G76 7AT UK; 5 Department of Psychiatry, College of medicine, Qassim University, 51452 Qassim, Kingdom of Saudi Arabia; 6 School of Pharmacy, College of Medical and Dental Sciences, University of Birmingham, Birmingham, B15 2TT UK

**Keywords:** Severe mental illness, Substance use disorders, Substance misuse, Substance abuse, Coexisting disorders, Dual diagnosis

## Abstract

**Objective:**

It is estimated that up to 75% of patients with severe mental illness (SMI) also have substance use disorder (SUD). The aim of this systematic review was to explore the scope, quality and inclusivity of international clinical guidelines on mental health and/or substance abuse in relation to diagnosis and treatment of co-existing disorders and considerations for wider social and contextual factors in treatment recommendations.

**Method:**

A protocol (PROSPERO CRD42020187094) driven systematic review was conducted. A systematic search was undertaken using six databases including MEDLINE, Cochrane Library, EMBASE, PsychInfo from 2010 till June 2020; and webpages of guideline bodies and professional societies. Guideline quality was assessed based on ‘Appraisal of Guidelines for Research & Evaluation II’ (AGREE II) tool. Data was extracted using a pre-piloted structured data extraction form and synthesized narratively. Reporting was based on PRISMA guideline.

**Result:**

A total of 12,644 records were identified. Of these, 21 guidelines were included in this review. Three of the included guidelines were related to coexisting disorders, 11 related to SMI, and 7 guidelines were related to SUD. Seven (out of 18) single disorder guidelines did not adequately recommend the importance of diagnosis or treatment of concurrent disorders despite their high co-prevalence. The majority of the guidelines (*n* = 15) lacked recommendations for medicines optimisation in accordance with concurrent disorders (SMI or SUD) such as in the context of drug interactions. Social cause and consequence of dual diagnosis such as homelessness and safeguarding and associated referral pathways were sparsely mentioned.

**Conclusion:**

Despite very high co-prevalence, clinical guidelines for SUD or SMI tend to have limited considerations for coexisting disorders in diagnosis, treatment and management. There is a need to improve the scope, quality and inclusivity of guidelines to offer person-centred and integrated care.

**Supplementary Information:**

The online version contains supplementary material available at 10.1186/s12888-021-03188-0.

## Background

It is estimated that up to 75% of patients with severe mental illness (SMI) also have substance use disorder (SUD) and about 60% of adults with SUD have at least one type of SMI [1–4], with one being either the cause or consequence of the other or various social issues leading to both issues at the same time [5, 6]. Genetic factors for such co-morbidity including variations in how people respond to treatments have also been suggested [7]. Coexisting disorders can result in greater incidence of adverse health outcomes, suicide, unplanned hospital admissions [2, 8–10] and early mortality [11–13]. Social consequences include violence, homelessness, involvement with criminal justice system, and relationship breakdowns have also been suggested [14–17]. For example, between a quarter and a third of prison populations in the Western countries are known to have a dual diagnosis [15, 18]. Involvement with criminal justice system is also known to adversely impact patient access to SMI and SUD services [19].

Assessment and treatment of patients in regard to dual diagnosis presents a challenge for care providers. Care providers can face challenges in managing psychiatric symptoms, substance craving, and social issues as a result of coexisting disorders [20]. In addition, fragmentation of care, for example, physical separation of services can result in barrier to access and provision of care [21–23]. Different opinion and divergent views of health care providers about treatment plan are also other known challenges [24, 25]. Parallel and separate care provided for each disorder within the same or different healthcare settings for patients with coexisting disorders are likely to be ineffective. This can lead to fragmentation of care, lack of timely access to treatment, withdrawal from treatment, physical multi-morbidity, and early deaths [9, 26, 27]. The advantage of considering both disorders together is that both SMI and SUD are simultaneously addressed and are given due attention [28]. However, practices are often patchy. Despite the known effectiveness of integrated treatment models for patients with coexisting disorders, integrated services availability remains sparse. A study conducted in the United States sampled programs from all over the US and showed that only 18% of addiction treatment and 9% of mental health programs had sufficient capacity to provide simultaneous services for patients with coexisting disorder [29].

A previous systematic review published in 2010 evaluated SMI and SUD guidelines to investigate whether or not they addressed co-occurring disorders [30]. The review considered guidelines published until 2007 and was limited to the inclusion of guidelines published in the National Guideline Clearinghouse database. Guidelines developed by the professional societies and clinical excellence committees are important decision tools that guide health care professionals’ care of their patients. Evidence-based guidelines allow practitioners to follow the best available evidence and also speeds up the adaptation of new treatment approaches. While practitioners may utilize professional judgements and conduct their own evidence search to inform person-centred care, guidelines are cornerstones in healthcare practice and adherence to clinical guidelines is often taken synonymous to evidence based practice [31]. The aim of this systematic review was to explore the scope, quality and inclusivity of international clinical guidelines on mental health and/or substance abuse in relation to diagnosis and treatment of such co-existing disorders and consideration of wider social and contextual issues in treatment recommendations.

## Methodology

### Protocol and registration

The study protocol registered in PROSPERO (CRD42020187094). The review was conducted as per PRISMA checklist and statement [32] (Electronic supplementary material 1).

### Criteria for considering guidelines for this review

The research for this review focused international guidelines which related to the assessment and treatment of either SUD, SMI or on concurrent disorders. The search was limited to guidelines published from 2010 until June 2020. To make sure that included guidelines represented current practice, guidelines published before 2010 were not considered. The search was restricted to guidelines published in the English language.

### Search and selection of guidelines

The research for guidelines was conducted using the following databases: MEDLINE, Cochrane Library, EMBASE, and PsychInfo, Google, Google scholar, Guideline Central; and national clinical guidelines and professional organizations’ web pages including National Institute for Health and Care Excellence (NICE) and the American Psychiatric Association (APA) .

The search terms used related to SUD and SMI MeSH terms (electronic supplemental material 2). The screening process was performed in three distinct stages including title, summary or abstract and full texts. The selection of guidelines done independently by two reviewers (RA and VP) and any discrepancies were resolved by consensus. We searched reference list of included guidelines to identify any further guidelines.

### Search definitions

We considered the Diagnostic and Statistical Manual of Mental Disorders (DSM-5) definition of, ‘substance use disorder’ which is a single term combines both abuse and dependence [33]. Such substances include legal drugs such as alcohol, illicit drugs such as heroin and cocaine, and prescription drugs such as oxycodone [34]. The SMIs considered in this review were psychosis and other associated types of schizophrenia, as well as bipolar disorder. The terms coexisting disorder, co-occurring disorder, or dual diagnosis are frequently used to describe the existence of both conditions of SMI and SUD simultaneously.

### Data extraction

After identification of eligible guidelines, data were extracted using a Microsoft Excel® spreadsheet. Data were extracted in relation to guideline characteristics, targeted patient population and health care providers, screening and management of co-existing disorders including recommendations for treatment adjustments and consideration of monitoring of physical health or drug interactions. Consideration of offending behavior, risks of homelessness, violence, and suicide were also extracted. Data extraction was done by two authors (RA and VP) in duplicate and independently and any disagreements were resolved by further discussion.

### Quality assessment

The included guidelines are appraised by using the Appraisal of Guidelines for Research & Evaluation II (AGREE II) tool. The assessment of each guideline is carried out by following the users’ instruction manual for AGREE II instruments [35]. The assessment for the following domains: ‘scope and purpose, stakeholder involvement, rigor of development, clarity of presentation, applicability, and editorial independence’ [36]. Each of the 23 items is scored 1 to 7 where 1 signals strong disagreement and 7 signals strong agreement and the final score is rated from 0 to 100%. In addition, there are two overall assessments of each guideline. The first one reflects the overall quality of each guideline. The second overall assessment allows assessment of whether or not the guideline is recommended for application in practice. Three distinct choices; namely, ‘Yes’, ‘Yes with modification’, or ‘No’ are utilized in relation to recommendation for use. Score sheet is demonstrated in Electronic supplemental material 3. Two reviewers independently assessed the included guidelines.

In order to calculate domain rate, the following equation from AGREE II users’ manual was used:

The rate of each domain = (total score of all items within the domain − lowest score of all items within the domain) / (highest score of all items within the domain − lowest score of all items within the domain) × 100.

A narrative synthesis was used to present the findings. Comparisons between guidelines are pre-identified in accordance with the particular objectives of the review.

## Results

### The search and selection of guidelines

**Fig. 1:**
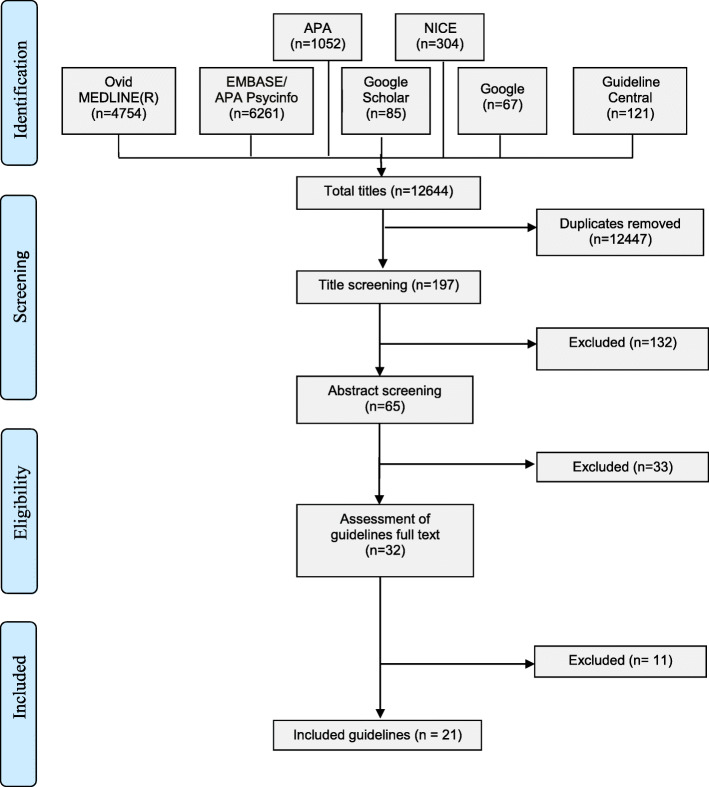
PRISMA* diagram of guidelines selection process

In total, 12,644 records were identified through the searching of various databases. After the exclusion of data de-duplication and both title and abstract screening, 32 guidelines were screened for eligibility. Twenty-one guidelines were included in this study (Fig. 1).

### General characteristics of the included guidelines

Of the 21 included, three guidelines related to coexisting disorders [37–39], seven guidelines related to SUD including alcohol use disorder and opioid disorder (Table 1) [40–46]. Eleven guidelines related to SMI (six of them were related to schizophrenia, and five of them were related to bipolar disorder) [47–57]. The aim of each guideline is illustrated in Table 1.

**Table 1:** General characteristic of the guidelines

Guideline title	Organization	Country	Publication year	Target disorders	Aim	For which patient population is this guideline intended for?	For which healthcare provider is this guideline intended for?	Clinical setting for which this is applicable
Coexisting severe mental illness (psychosis) and substance misuse: assessment and management in healthcare settings [37]	NICE	UK	2011	Psychosis + SUD	To provide diagnosis and treatment recommendations for both disorders.	For all patients above 14 years old with both disorders.	For professionals who provide care in all clinical settings.	All clinical settings and medical services that commissioned by NHS
Coexisting severe mental illness and substance misuse: community health and social care services overview [38]	NICE	UK	2016	Psychosis + SUD	To offer a number of integrated services to meet people’s requirements and solve other related problems, such as lack of housing and joblessness.	For patients above 14 years old with both disorders.	All professionals and commissioners, Workers who have direct contact with patients, The criminal justice system, Voluntary organizations and other third-party sectors, Targeted patients and their families and carers.	Community settings
Guidelines on the management of cooccurring alcohol and other drug and mental health conditions in alcohol and other drug treatment settings [39]	Australian government	Australia	2016	Co-occurring alcohol and other drug and mental health conditions	To provide directives in relation to the management of AOD and co-occurring mental health conditions.	Patients with AOD and co-occurring mental health conditions.	AOD workers, including nurses, medical practitioners, psychiatrists, psychologists, counsellors, social workers, and other AOD workers.	AOD treatment settings
Management of schizophrenia [47]	SIGN	UK	2013	Schizophrenia	To provide recommendations for managing schizophrenia.	Adults with schizophrenia	Healthcare providers	Not mentioned specifically
Psychosis and schizophrenia in adults: prevention and management [48]	NICE	UK	2014	Psychosis and schizophrenia	To provide diagnosis and treatment recommendations psychosis and schizophrenia.	Adults with psychosis and schizophrenia	Health care providers who provide services in primary, community, secondary and tertiary clinical settings.	All clinical settings and medical services that commissioned by NHS
Guidelines for Biological Treatment of Schizophrenia. Part 3: Update 2015 Management of special circumstances: Depression, Suicidality, substance use disorders and pregnancy and lactation [49]	WFSBP	International	2015	Schizophrenia	To issue guidelines relating to the management of schizophrenia and the assessment of pharmacological agents.	Patients with schizophrenia.	Physicians	Not mentioned specifically
Clinical practice guidelines for the management of schizophrenia and related disorders [50]	RANZCP	Australia and New Zealand	2016	Schizophrenia and related disorders	To provide guidance for the treatment of schizophrenia and to provide care for schizophrenic patients.	For patients with schizophrenia and related disorders.	Clinicians such as psychiatrists and GPs, psychiatry trainees, mental health nurses, clinicians who have contact with this patient group, and policymakers.	Not mentioned specifically
Evidence-based guidelines for the pharmacological treatment of schizophrenia [51]	BAP	UK	2019	Schizophrenia	To provide recommendations for the management of schizophrenia.	Patients with schizophrenia	Clinicians	Not mentioned specifically
Practice guideline for the treatment of patients with schizophrenia [52]	APA	US	2020	Schizophrenia	To help clinicians optimize care for their patients and improve quality of care.	Patients with schizophrenia	Clinicians	Not mentioned specifically
Management of Bipolar Disorder in Adults (BD) [53]	VA/DoD	US	2010	Bipolar disorder	To manage patients with bipolar disorder.	People aged 18 years old and older with bipolar disorder.	Healthcare professionals	Not mentioned specifically
Bipolar disorder [54]	Singapore MOH	Singapore	2011	Bipolar disorder	To manage patients with bipolar disorder.	Older patients with bipolar disorder	GP and clinicians	Not mentioned specifically
The assessment and management of bipolar disorder in adults, children and young people in primary and secondary care [55]	NICE	UK	2014	Bipolar disorders	To manage patients with bipolar disorder.	Children, young adults (aged above 13 years old), and adults.	Professionals who provide care in all clinical settings.	All clinical settings and medical services that commissioned by NHS
Evidence-based guidelines for treating bipolar disorder [56]	BAP	UK	2016	Bipolar disorder	To assess and manage patients with bipolar disorder.	Patients with bipolar disorder.	Practitioners	Not mentioned specifically
Guidelines for the management of patients with bipolar disorder [57]	CANMAT and ISBD	Canada	2018	Bipolar disorder	To manage patients with bipolar disorder.	Patients with bipolar disorder	Psychiatrists and primary care providers	Not mentioned specifically
Evidence-based guidelines for the pharmacological management of substance abuse, harmful use, addiction and comorbidity [40]	BAP	UK	2012	SUD	To provide treatment recommendations in order to help clinicians in prescribing medication for patients with SUD alone and those with psychiatric comorbidities.	Young adults and adults with SUD.	Clinicians such as psychiatrists and GPs, professionals in this field, non-specialists, patients and their families.	Not mentioned specifically
Guidelines for biological treatment of substance use and related disorders, part 1: Alcoholism, first revision [41]	WFSBP	International	2017	Substance use and related disorders	To provide recommendations for the treatment of AUD that help clinicians in clinical decision making and subsequently improvement of care	Adult with AUD	Professionals who provide care for patients with AUD.	Not mentioned specifically
Drug misuse and dependence UK guidelines on clinical management [42]	gov.uk	UK	2017	Substance misuse	To provide guidance on managing drug abuse and dependency in the UK.	Drug misusers	Healthcare professionals, Regulatory bodies, Targeted patients, and their families and carers.	Drug misuse services
German Guidelines on Screening, Diagnosis and Treatment of Alcohol Use Disorders [43]	DGPPN and DG-Sucht	Germany	2017	Alcohol use disorder	To provide screening, diagnosis, and treatment recommendations for patients with alcohol misuse disorder.	Patients with alcohol misuse disorder and comorbidity psychiatric disorders.	Clinicians	In- and outpatient settings
Alcohol use disorders: diagnosis, assessment and management of harmful drinking and alcohol dependence [44]	NICE	UK	2011	Alcohol use disorder	To provide recommendations for managing patients with alcohol misuse disorder.	Younger children and young adults 10–17 years old with alcohol use disorder.	Professionals who provide care in all clinical settings.	All clinical settings and medical services that commissioned by NHS
Practice guideline for the Pharmacological Treatment of Patients with Alcohol Use Disorder [45]	APA	US	2018	Alcohol use disorder	To provide recommendations that help in improving the quality of care and quality of life for patients with AUD.	Patients with AUD	Clinicians	Not mentioned specifically
National Practice Guideline for the Use of Medications in the Treatment of Addiction Involving Opioid Use [46]	ASAM	US	2015	Opioid use disorder	To provide recommendations for managing patients with opioid use disorder.	Patients with opioid use disorder	Physicians; other healthcare providers, medical educators, trainee; and clinical care managers.	Not mentioned specifically

*AOD* Alcohol and other drug, *APA* American Psychiatric Association, *ASAM* American society of addiction medicine, *AUD* alcohol use disorder, *BAP* British Association of psychopharmacology, *CANMAT and ISBD* Canadian Network for Mood and Anxiety Treatments and International Society for Bipolar Disorders, *DGPPN and DG-Sucht* German Association for Psychiatry, Psychotherapy, and Psychosomatics and the German Association for Addiction Research and Therapy, *gov.UK* United Kingdom guidelines on clinical management, *NICE* National Institute for Health and Care Excellence, *NIH* National health service, *RANZCP* Royal Australian and New Zealand College of Psychiatrists, *SIGN* Scottish Intercollegiate Guidelines Network, *Singapore MOH* Singapore Ministry of Health, *SMI* Severe mental illness, *SUD* Substance use disorder, *UK* United Kingdom, *US* United States, *VA/DoD* Department of Veterans Affairs and The Department of Defense, *WFSBP* World Federation of Societies of Biological Psychiatry

Most of the included guidelines were produced by NICE in England (*n* = 5), followed by guidelines produced by British Association of Psychopharmacology in the UK (*n* = 3). Two of the included guidelines were published by APA in the USA, two of them were produced by the World Federation of Societies of Biological Psychiatry (WFSBP) which developed by a group of experts from different countries, and nine guidelines were published by government departments of health [39, 42, 43, 46, 47, 50, 53, 54, 57] (Table 1).

### Quality assessment of guidelines

**Fig. 2:**
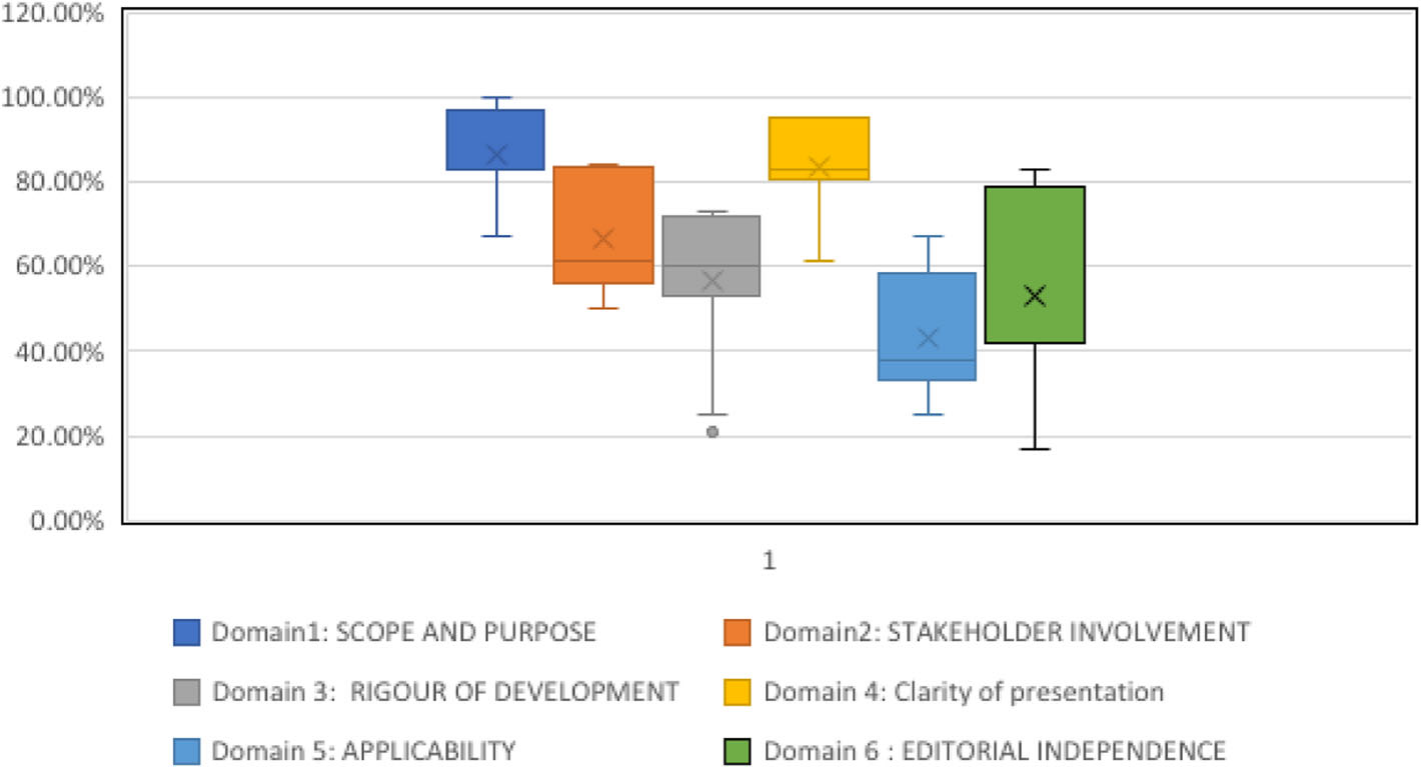
Combined AGREE II assessment of guidelines

The scores of each guideline against the criteria of the AGREE II tool are displayed in Table 2. In terms of ‘scope and purpose’, first domain had the highest domain score. Only four guidelines scored below 80% [39, 46, 54, 57] (Table 2). In the second domain, ‘stakeholder involvement’, the guidelines that were developed by NICE and Scottish Intercollegiate Guidelines Network (SIGN) demonstrated the highest score; 84 and 83%, respectively [37, 38, 44, 47, 48, 55] (Table 2). The ‘Rigour of development’ domain scores were generally low (Fig. 2). Fifteen out of 21 included guidelines rated below 70% (Table 2). Most of the guidelines scored higher in ‘Clarity of presentation’ domain (Fig. 2). The guidelines that were developed by NICE and SIGN obtained the highest scores [37, 38, 44, 47, 48, 55] (Table 2). Figure 2 shows that the ‘Applicability’ domain has the lowest domain score. Fifteen guidelines were graded below 50% (Table 2). With regard to the ‘Editorial independence’ domain, the highest score was reported with the NICE guidelines, this being 83%. The rest of the included guidelines were graded below 80% (Table 2, Fig. 2).

**Table 2:** Quality assessment of guidelines

Guideline	Domain1: Scope and purpose	Domain2: Stakeholder involvement	Domain 3: Rigour of development	Domain 4: Clarity of presentation	Domain 5: Applicability	Domain 6: Editorial independence	Overall quality	Recommendation of use
NICE 2011 [37]	100.00%	84.00%	73.00%	95.00%	67.00%	83.00%	7	Recommended
NICE 2014 [55]	100.00%	84.00%	73.00%	95.00%	67.00%	83.00%	7	Recommended
RANZCP 2016 [50]	83.00%	72.00%	35.00%	83.00%	33.00%	42.00%	4	Recommended with modification
BAP 2012 [40]	83.00%	61.00%	54.00%	83.00%	42.00%	42.00%	5	Recommended with modification
WFSBP 2017 [41]	83.00%	50.00%	60.00%	67.00%	25.00%	42.00%	4	Recommended with modification
gov.uk 2017 [42]	94.00%	67.00%	60.00%	72.00%	33.00%	33.00%	7	Recommended
DGPPN and DG-Sucht 2017 [43]	83.00%	56.00%	63.00%	78.00%	33.00%	50.00%	4	Recommended with modification
NICE 2011 [44]	100.00%	84.00%	73.00%	95.00%	67.00%	83.00%	7	Recommended
ASAM 2015 [46]	67.00%	72.00%	52.00%	83.00%	38.00%	58.00%	5	Recommended with modification
APA 2018 [45]	89.00%	56.00%	65.00%	83.00%	42.00%	75.00%	6	Recommended
Singapore MOH 2011 [54]	67.00%	56.00%	25.00%	89.00%	38.00%	17.00%	3	Not recommended
VA/DoD 2010 [53]	83.00%	56.00%	58.00%	83.00%	38.00%	17.00%	4	Recommended with modification
CANMAT & ISBD 2018 [57]	67.00%	61.00%	31.00%	61.00%	29.00%	42.00%	3	Not recommended
SIGN 2013 [47]	94.00%	83.00%	71.00%	95.00%	50.00%	50.00%	7	Recommended
WFSBP 2015 [49]	83.00%	50.00%	60.00%	67.00%	25.00%	42.00%	4	Recommended with modification
NICE 2016 [38]	100.00%	84.00%	73.00%	95.00%	67.00%	83.00%	7	Recommended
NICE 2014 [48]	100.00%	84.00%	73.00%	95.00%	67.00%	83.00%	7	Recommended
BAP 2019 [51]	83.00%	61.00%	54.00%	83.00%	33.00%	42.00%	5	Recommended with modification
BAP 2016 [56]	83.00%	61.00%	54.00%	83.00%	33.00%	42.00%	5	Recommended with modification
APA 2020 [52]	89.00%	56.00%	65.00%	83.00%	42.00%	75.00%	6	Recommended
Australian government 2016 [39]	78.00%	56.00%	21.00%	89.00%	38.00%	25.00%	3	Not recommended

*APA* American Psychiatric Association, *ASAM* American society of addiction medicine, *BAP* British Association of psychopharmacology, *CANMAT and ISBD* Canadian Network for Mood and Anxiety Treatments and International Society for Bipolar Disorders, *gov.UK* United Kingdom guidelines on clinical management, *DGPPN and DG-Sucht* German Association for Psychiatry, Psychotherapy, and Psychosomatics and the German Association for Addiction Research and Therapy, *NICE* National Institute for Health and Care Excellence, *RANZCP* Royal Australian and New Zealand College of Psychiatrists, *SIGN* Scottish Intercollegiate Guidelines Network, *Singapore MOH* Singapore Ministry of Health, *VA/DoD* Department of Veterans Affairs and The Department of Defense, *WFSBP* World Federation of Societies of Biological Psychiatry

### Assessment of concurrent problems

All of the included coexisting disorders guidelines emphasized that a comprehensive assessment should be carried out for patients with either SMI or SUD for dual diagnosis [37–39]. However, five out of eleven (45%) SMI guidelines did not highlight the assessment of coexisting disorders [47, 48, 54, 55, 57]. In addition, one SUD guidelines (14%) did not highlight the assessment of coexisting disorders [41] (Table 3).

**Table 3:** Consideration of concurrent problems

Guideline	Is the link between mental health and misuse of substances mentioned as part of the background?	Does the guideline mention that either SMI or SUD can worsen the outcome of another?	Does the guideline provide recommendations about Screening/Assessment for coexisting disorders?	Does the guideline mention the competence of healthcare professionals in recognition the existence of the other comorbidity (i.e. either substance misuse or mental health problems)?	Does the guideline requests healthcare professionals to seek advice or training from the other service i.e. training from substance misuse service staff to staff in mental health services?	Does the guideline specifically mention not to exclude patients who misuse substance from age-appropriate treatment settings of mental illness due to use of substances?	Does the guideline specifically mention not to exclude patients who have mental illness from age-appropriate treatment setting of substance misuse due to mental health problems?	Who refers patients to a mental health setting or to the substance misuse/alcohol misuse services?	If a guideline is for mental health, does it mention not to discharge patients from inpatient services because of their substance misuse?
NICE 2011 (coexisting disorders) [37]	Yes	Yes	Mentioned. Suspected patients should be asked about any drugs and alcohol drinking including: its type, quantity, frequency, route of administration, and duration of use.	Yes	Yes	Yes	Yes	Mentioned. All staff who have direct contact with patients, including professionals in primary care and educational settings.	Yes
NICE 2016 (coexisting disorders) [38]	Yes	Yes	Mentioned. A Full evaluation for suspected patients.	No	Yes	Yes	No	Mentioned. All staff who have direct contact with patients, including professionals in primary care and educational settings.	No
Australian government 2016 [39]	Yes	Yes	Mentioned. After abstinence, a full assessment of the patient should ideally occur.	Yes	Yes	No	No	Not mentioned	Not applicable
SIGN 2013 [47]	Yes	Yes	Not mentioned	Yes	Yes	Yes	No	Not mentioned	No
NICE 2014 [48]	Yes	Yes	Not mentioned	No	Yes	No	No	Mentioned. Primary healthcare professionals	No
WFSBP 2015 [49]	Yes	Yes	Mentioned. Detailed assessment of substance use disorder should be obtained.	No	No	No	No	Not mentioned	No
RANZCP 2016 [50]	Yes	Yes	Mentioned. Any suspicions regarding the use of stimulant drugs should be raised if there are recurrent episodes of psychosis.	Yes	Yes	No	No	Mentioned. Health care professionals and any other professionals involved in providing care for patients, such as GPs and social counsellors.	No
BAP 2019 [51]	Yes	Yes	Mentioned. A detailed assessment of substance use disorder should be obtained.	No	No	No	No	Not mentioned	No
APA 2020 [52]	Yes	Yes	Mentioned. Any initial assessment of a patients with a possible psychotic disorder should include an assessment of their tobacco use and other substance misuse.	Yes	Yes	No	No	Not mentioned	No
VA/DoD 2010 [53]	Yes	Yes	Mentioned. A complete clinical assessment should be obtained.	No	No	No	No	Not mentioned	Yes
Singapore MOH 2011 [54]	Yes	Yes	Not mentioned	No	No	No	No	Not mentioned	No
NICE 2014 [55]	Yes	Yes	Not mentioned	No	Yes	No	No	Mentioned. Primary healthcare professionals	No
BAP 2016 [56]	No	Yes	Mentioned. The clinician should assess to what extent does substance misuse contribute to bipolar symptoms.	No	Yes	No	No	Not mentioned	No
CANMAT and ISBD 2018 [57]	Yes	Yes	Not mentioned	Yes	No	No	No	Not mentioned	No
BAP 2012 [40]	Yes	Yes	Mentioned. Substance history, family history, urinalysis and blood tests, as well as an assessment of psychiatric disorder onset, and the misuse of substances should be carried out.	No	No	No	No	Not mentioned	Not applicable
WFSBP 2017 [41]	Yes	Yes	Not mentioned	No	No	No	No	Not mentioned	Not applicable
gov.uk 2017 [42]	Yes	Yes	Mentioned. Identifying any current or previous psychological problems	Yes	Yes	Yes	No	Mentioned. GPs	Not applicable
DGPPN and DG-Sucht 2017 [43]	Yes	Yes	Mentioned. The assessment process derived from alcohol use disorder identification test guidelines	No	No	No	No	Not mentioned	Yes
NICE 2011 [44]	Yes	Yes	Mentioned. Patients should be referred to a psychiatrist for effective assessment and treatment.	Yes	Yes	No	No	Mentioned. Whole range of healthcare such as a GP.	Not applicable
APA 2018 [45]	Yes	Yes	Mentioned. Patients should be assessed for alcohol use disorder and comorbid mental health disorder.	No	No	No	No	Not mentioned	No
ASAM 2015 [46]	Yes	Yes	Mentioned. A comprehensive assessment of the patient and any ideas related to suicide should be evaluated. The patient’s full medical history and a physical examination should also be obtained.	Yes	Yes	No	No	Not mentioned	Not applicable

*AOD* Alcohol and other drug, *APA* American Psychiatric Association, *ASAM* American society of addiction medicine, *AUD* alcohol use disorder, *BAP* British Association of psychopharmacology, *CANMAT and ISBD* Canadian Network for Mood and Anxiety Treatments and International Society for Bipolar Disorders, *gov.UK* United Kingdom guidelines on clinical management, *GPs* General practitioners, *DGPPN and DG-Sucht* German Association for Psychiatry, Psychotherapy, and Psychosomatics and the German Association for Addiction Research and Therapy, *NICE* National Institute for Health and Care Excellence, *RANZCP* Royal Australian and New Zealand College of Psychiatrists, *SIGN* Scottish Intercollegiate Guidelines Network, *Singapore MOH* Singapore Ministry of Health, *SMI* Severe mental illness, *SUD* Substance use disorder, *UK* United Kingdom, *US* United States, *VA/DoD* Department of Veterans Affairs and The Department of Defense, *WFSBP* World Federation of Societies of Biological Psychiatry

Three guidelines explicitly stated that patients with SMI with coexisting SUD who completed their SMI treatment course should stay in the hospital to avoid exacerbation of psychotic symptoms and future risk due to substance abuse not be discharged from a healthcare setting due to their substance abuse [37, 43, 53]. Of the SMI guidelines, four guidelines highlighted the competency need of healthcare providers in each health care setting to consider for the co-existing disorders [47, 50, 52, 57]. Three out of seven SUD guidelines similarly covered competency aspects [42, 44, 46]. All coexisting disorder guidelines requested healthcare providers to gain training and expertise from other specialist staff in regards to either SMI or SUD [37–39] (Table 3).

### Treatment of coexisting disorders

All of the guidelines related to SMI or coexisting disorders described the importance of screening and/or treatment for both problems simultaneously [37–39]. Three (27%) SMI guidelines stipulated SUD clinical guidelines and vice versa when recommending treatment of the other co-existing disorder (Table 4) [53, 55, 56]. One SUD guideline (14%) [45] however, did not explicitly provide recommendation regarding treatment of both disorders.

**Table 4:** Consideration of treatment adjustments

Guideline	Does the recommendation address the management of coexisting disorders?	What treatment adjustment should be considered? (such as a change of antipsychotic medication in patients who have alcohol use disorder)	Recommendation for monitoring of physical health	Recommendation about drug interactions	Psychological and psychosocial interventions
NICE 2011 (coexisting disorders) [37]	No recommendations regarding the benefits of one antipsychotic over another are given. Refers the reader to the NICE guidelines for related disorders.	Mentioned. Use of long-acting injectable antipsychotic medication	Mentioned	Mentioned. Substance misuse practically alcohol may affect the metabolism of medication	Mentioned
NICE 2016 (coexisting disorders) [38]	Not mentioned. Refers the reader to a NICE guideline for the management of coexisting disorders.	Not mentioned	Mentioned	Not included	Not mentioned
Australian government 2016 [39]	Mentioned. Detailed treatment plan for both psychosis and bipolar disorder are provided	Not mentioned	Mentioned	Included	mentioned
SIGN 2013 [47]	Mentioned. The treatment of both disorders requires a joint consultative approach between the services provided from both mental health and substance use settings.	Not mentioned	Mentioned	Not included	Mentioned
NICE 2014 [48]	Mentioned. Monitoring for coexisting conditions particularly in the early phases of treatment.	Not mentioned	Mentioned	Not included	Mentioned
WFSBP 2015 [49]	Mentioned. Consider the addition of clozapine for coexisting disorder.	Mentioned. Patients with a history of non-adherence to their medication should be treated with long-acting depot formulations of antipsychotic medications.	Not mentioned	Not included	Mentioned
RANZCP 2016 [50]	Mentioned. Treatment of comorbid substance use. Urine or saliva drug testing for the presence of substance misuse should also be employed.	Not mentioned	Mentioned	Not included	Mentioned
BAP 2019 [51]	Mentioned. Optimization of antipsychotic medication and one should consider the addition of clozapine for the patients with dual diagnosis.	Not mentioned	Mentioned	Included	Mentioned
APA 2020 [52]	Mentioned. Treatment for both disorders should be provided by the same clinician team. However, if an integrated treatment is unavailable, the treatment plan should address both disorders with communication and collaboration among the clinicians treating the patient.	Not mentioned	Mentioned	Included	Mentioned
VA/DoD 2010 [53]	For the management of substance misuse, the reader should refer to the VA/DoD guideline for other related disorders. Treatment of bipolar disorder should be based on this guideline.	Not mentioned	Mentioned	Included	Mentioned
Singapore MOH 2011 [54]	Mentioned. Patients with both addiction and bipolar disorders should be treated.	Not mentioned	Not mentioned	Not included	Mentioned
NICE 2014 [55]	The reader should refer to the NICE guideline for other related disorders. Moreover, bipolar disorder treatment should be in accordance with this guideline.	Not mentioned	Mentioned	Included	Mentioned
BAP 2016 [56]	For alcohol use disorder, this guideline refers the reader to another BAP guideline. The practitioner should assess to what extent substance misuse contributes to bipolar disorder symptom.	Not mentioned	Mentioned	Included	Mentioned
CANMAT and ISBD 2018 [57]	Mentioned. For patients with both bipolar disorder and substance misuse, lithium can reduce using of substance. Patients with both bipolar disorder and substance misuse may benefit from the use of N-acetylcysteine.	Mentioned. Reduce bipolar disorder symptoms with olanzapine. Reduce cravings for alcohol and cocaine use with aripiprazole.	Mentioned	Not included	Mentioned
BAP 2012 [40]	Treatment of bipolar disorder as recommended in other guidelines and the impact of harmful substance use should be assessed.	Mentioned. Add sodium valproate for bipolar disorder patients who are on lithium only, and limit alcohol drinking with Naltrexone. Clozapine should be considered in patients with both schizophrenia and substance misuse.	Mentioned	Not included	Not mentioned
WFSBP 2017 [41]	It is difficult to provide treatment recommendations for managing patients with both schizophrenia and coexisting alcohol use disorder.	Mentioned. Suggest the use of second generation antipsychotics for managing patients with both schizophrenia and coexisting alcohol use disorder. However, evidence recommends the use of clozapine.	Not mentioned	Not included	Mentioned
gov.uk 2017 [42]	Mentioned. Dual focused treatments	Not mentioned	Mentioned	Included	Mentioned
DGPPN and DG-Sucht 2017 [43]	Mentioned. Pharmacological treatment should be based on schizophrenia guidelines.	Mentioned. Treatment of patients with schizophrenia and comorbid alcohol use disorder with atypical antipsychotics (AAP).	Not mentioned	Not included	Mentioned
NICE 2011 [44]	Mentioned. For the treatment of comorbid mental health disorders, the reader is referred to the other related disorder’s NICE guideline.	Not mentioned	Mentioned	Included	Mentioned
APA 2018 [45]	Not mentioned.	Not mentioned	Not mentioned	Not included	Not mentioned
ASAM 2015 [46]	Mentioned. Use of mood stabilizers for the treatment of patients with bipolar disorder. Patients with schizophrenia should be treated with suitable antipsychotic therapy along with treatment of opioid use disorder. Patients with a history of non-adherence to their medication should be treated with long-acting depot formulations of antipsychotic medications. Methadone, buprenorphine, or naltrexone for mental status stabilization.	Not mentioned	Mentioned	Included	Mentioned

*AOD* Alcohol and other drug, *APA* American Psychiatric Association, *ASAM* American society of addiction medicine, *AUD* alcohol use disorder, *BAP* British Association of psychopharmacology, *CANMAT and ISBD* Canadian Network for Mood and Anxiety Treatments and International Society for Bipolar Disorders, *gov.UK* United Kingdom guidelines on clinical management, *DGPPN and DG-Sucht* German Association for Psychiatry, Psychotherapy, and Psychosomatics and the German Association for Addiction Research and Therapy, *NICE* National Institute for Health and Care Excellence, *RANZCP* Royal Australian and New Zealand College of Psychiatrists, *SIGN* Scottish Intercollegiate Guidelines Network, *Singapore MOH* Singapore Ministry of Health, *SMI* Severe mental illness, *SUD* Substance use disorder, *UK* United Kingdom, *US* United States, *VA/DoD* Department of Veterans Affairs and The Department of Defense, *WFSBP* World Federation of Societies of Biological Psychiatry

Only two out of the 11 (18%) SMI guidelines mentioned recommendation about treatment adjustments when considering dual diagnosis and treatment [49, 57]. Similarly, only three of the seven (43%) SUD guidelines mentioned recommendation about treatment adjustment [40, 41, 43] (Table 4). Examples of treatment adjustments included recommendation for the use of long-acting injectable antipsychotic medication in cases where there was a history of non-adherence to medication in place of regular antipsychotic medication [49]. Only one (33%) guideline of the coexisting disorders guidelines recommended the use of long-acting injectable antipsychotic medication accordingly [37]. Two (67%) of the guidelines related to coexisting disorders [37, 39], five (45%) of SMI guidelines [51–53, 55, 56] and three (43%) of the SUD guidelines [42, 44, 46] considered potential drug interaction in patients with SMI and coexisting SUD. For example, the NICE (2011) guideline recommends that caution be exercised during the prescribing of medication for patients demonstrating substance abuse particularly that of alcohol, since alcohol will affect the metabolism of other medications and either diminish their efficacy or increase the risk of side effects [37] (Table 4).

Importance of physical health monitoring were described by all guidelines related to coexisting disorders, nine (82%) SMI guidelines, and four of the seven (57%) SUD guidelines. These included monitoring and management of diabetes mellitus and hyperlipidemia (Table 4).

### Care pathway and integrated care provision

All of the coexisting disorders guidelines, seven (64%) of the SMI guidelines, and three (43%) of SUD guidelines mentioned the importance of continuity of care. For example, the Australian government guideline advised that it is important to develop systems in order to facilitate the transition of patients with coexisting disorders by providing them with much-needed services and helping them to address their complex needs [39] (Table 5).

**Table 5:** Care pathway and integrated care provision

Guideline	Does the guideline mention continuity of care i.e. importance of same health or key worker in managing the substance misuse or mental health/ continuity of care?	Where should integrated services be provided	Is there a mention of the role of emergency department or A&E and what they should do if patients present there?
NICE 2011 (coexisting disorders) [37]	Yes	Secondary care mental health services, CAMHS	Yes
NICE 2016 (coexisting disorders) [38]	Yes	Mental health services	No
Australian government 2016 [39]	Yes	AOD settings	No
SIGN 2013 [47]	No	Not mentioned	No
NICE 2014 [48]	Yes	secondary care settings	No
WFSBP 2015 [49]	No	Not mentioned	No
RANZCP 2016 [50]	Mentions continuity but not link key worker	Dual diagnosis service	No
BAP 2019 [51]	No	Not mentioned	No
APA 2020 [52]	Yes	Not mentioned	No
VA/DoD 2010 [53]	Yes	Urgent/emergent mental health settings	No
Singapore MOH 2011 [54]	Yes	In an integrated specialist treatment centre.	No
NICE 2014 [55]	Yes	Not mentioned	No
BAP 2016 [56]	No	Not mentioned	No
CANMAT and ISBD 2018 [57]	Yes	inpatient hospital or community residential treatment	No
BAP 2012 [40]	No	Not mentioned	No
WFSBP 2017 [41]	No	Not mentioned	No
gov.uk 2017 [42]	Yes	In drug misuse services and mental health services	Yes
DGPPN and DG-Sucht 2017 [43]	No	Inpatient treatment	No
NICE 2011 [44]	Yes	Not mentioned	Yes
APA 2018 [45]	Yes	Not mentioned	No
ASAM 2015 [46]	No	Hospitals	Yes

*AOD* Alcohol and other drug, *APA* American Psychiatric Association, *ASAM* American society of addiction medicine, *AUD* alcohol use disorder, *BAP* British Association of psychopharmacology, *CANMAT and ISBD* Canadian Network for Mood and Anxiety Treatments and International Society for Bipolar Disorders, *gov.UK* United Kingdom guidelines on clinical management, *DGPPN and DG-Sucht* German Association for Psychiatry, Psychotherapy, and Psychosomatics and the German Association for Addiction Research and Therapy, *NICE* National Institute for Health and Care Excellence, *RANZCP* Royal Australian and New Zealand College of Psychiatrists, *SIGN* Scottish Intercollegiate Guidelines Network, *Singapore MOH* Singapore Ministry of Health, *SMI* Severe mental illness, *SUD* Substance use disorder, *UK* United Kingdom, *US* United States, *VA/DoD* Department of Veterans Affairs and The Department of Defense, *WFSBP* World Federation of Societies of Biological Psychiatry

Only one (33%) of the guidelines pertaining to coexisting disorders mentioned that healthcare providers in the emergency department should regularly ask patients about any potential substance abuse [37]. Three (43%) of the guidelines related to SUD mentioned the role of the emergency department [42, 44, 46]. Such consideration was missing from SMI guidelines (Table 5).

### Equity consideration and person-centered care

Three guidelines pertaining to coexisting disorders, ten (91%) SMI guidelines, and six (86%) SUD guidelines described the essential role played by ‘significant others’ such as families and carers and encouraged their involvement along with any integrated care plans provided to patients (Table 6). All of the three guidelines pertaining to coexisting disorders were explicit in reporting the need for assessment of any children cared for by patients with both disorders, according to safeguarding procedures. However, only three (27%) of the SMI guidelines and two (29%) of the SUD guidelines provided recommendations about children cared for by patients with both disorders (Table 6).

**Table 6:** Equity considerations and person-centered care

Guideline	Does the guideline mention the importance of involving family and carers?	Dose the guideline mention children cared for by patient with mental health conditions or substance misuse?	Does the guideline mention the importance of engaging with various ethnicities and cultural needs?	Does the guideline mention allaying patient fear about being detained or forcefully put into care or rehabilitation?	Is there consideration for people with physical, sensory or learning disabilities in the guideline?
NICE 2011 (coexisting disorders) [37]	Yes	Yes	Yes	Yes	Yes
NICE 2016 (coexisting disorders) [38]	Yes	Yes	Yes	No	Yes
Australian government 2016 [39]	Yes	Yes	Yes	No	Yes
SIGN 2013 [47]	Yes	Yes	Yes	No	Yes
NICE 2014 [48]	Yes	No	Yes	No	Yes
WFSBP 2015 [49]	Yes	No	No	No	No
RANZCP 2016 [50]	Yes	No	Yes	No	No
BAP 2019 [51]	Yes	No	No	No	Yes
APA 2020 [52]	Yes	No	Yes	No	Yes
VA/DoD 2010 [53]	Yes	No	No	No	Yes
Singapore MOH 2011 [54]	No	No	No	No	No
NICE 2014 [55]	Yes	Yes	Yes	No	Yes
BAP 2016 [56]	Yes	Yes	No	No	No
CANMAT and ISBD 2018 [57]	Yes	No	No	No	Yes
BAP 2012 [40]	Yes	No	No	No	No
WFSBP 2017 [41]	Yes	No	No	No	No
gov.uk 2017 [42]	Yes	Yes	Yes	No	Yes
DGPPN and DG-Sucht 2017 [43]	No	No	No	No	No
NICE 2011 [44]	Yes	Yes	Yes	No	Yes
APA 2018 [45]	Yes	No	No	No	Yes
ASAM 2015 [46]	Yes	No	No	No	Yes

*AOD* Alcohol and other drug, *APA* American Psychiatric Association, *ASAM* American society of addiction medicine, *AUD* alcohol use disorder, *BAP* British Association of psychopharmacology, *CANMAT and ISBD* Canadian Network for Mood and Anxiety Treatments and International Society for Bipolar Disorders, *gov.UK* United Kingdom guidelines on clinical management, *DGPPN and DG-Sucht* German Association for Psychiatry, Psychotherapy, and Psychosomatics and the German Association for Addiction Research and Therapy, *NICE* National Institute for Health and Care Excellence, *RANZCP* Royal Australian and New Zealand College of Psychiatrists, *SIGN* Scottish Intercollegiate Guidelines Network, *Singapore MOH* Singapore Ministry of Health, *SMI* Severe mental illness, *SUD* Substance use disorder, *UK* United Kingdom, *US* United States, *VA/DoD* Department of Veterans Affairs and The Department of Defense, *WFSBP* World Federation of Societies of Biological Psychiatry

All of the guidelines pertaining to coexisting disorders, five (45%) of the SMI guidelines, and two (29%) of the SUD guidelines mentioned the importance of ensuring that healthcare providers who provide care to patients with coexisting disorders should engage with patients from different ethnicities and cultural backgrounds (Table 6). Only the NICE 2011 offered advice to healthcare providers to solve access to care issues in patients [37] (Table 6).

### Consideration of multiple social disadvantage

All of the guidelines pertaining to coexisting disorders, nine (82%) of the SMI guidelines, and five (71%) of the SUD guidelines considered the assessment of risks of violence, suicide, and self-harm (Table 7). Two (67%) of the guidelines pertaining to coexisting disorders highlighted the risk of certain getting involved with criminal justice system and the importance of prevention actions [37, 38]. Only the SMI guideline by Royal Australian and New Zealand College of Psychiatrists (RANZCP) [50] and three (45%) of the SUD guidelines [42, 44, 46] highlighted the risk of patients being registered in the criminal justice system (Table 7).

**Table 7:** Inclusivity in relation to consideration of homelessness and contextual factors

Guideline	Does the guideline mention that concurrent problems can increase risk of self-harm, suicide, violence, injury or offending behaviour?	Is the risk of criminal justice system/offending/prison for those affected mentioned?	Does the guideline recommend providing health care for prison offender in rehabilitation centre	Is the risk of homelessness for those affected mentioned?	Does the guideline provide suggestions for healthcare professionals to refer patients to housing assistance or homelessness services if patients are found at risk of homelessness	Does the screening mentions patient history of sexual or other forms of abuse?	Is there mention of or consideration about stigma and discrimination in healthcare setting?	Does the guideline mention the importance of working with voluntary, charity or No?
NICE 2011 (coexisting disorders) [37]	Yes	Yes	Yes, in case of diverted treatment	Yes	Yes	Yes	Yes	Yes
NICE 2016 (coexisting disorders) [38]	Yes	Yes	No	Yes	Yes	No	Yes	Yes
Australian government 2016 [39]	Yes	No	No	Yes	No	Yes	Yes	No
SIGN 2013 [47]	Yes	No	No	Yes	Yes	No	No	No
NICE 2014 [48]	No	No	No	No	No	No	Yes	Yes
WFSBP 2015 [49]	Yes	No	No	Yes	Yes	No	No	No
RANZCP 2016 [50]	Yes	Yes	No	Yes	Yes	No	Yes	Yes
BAP 2019 [51]	No	No	No	No	No	No	No	No
APA 2020 [52]	Yes	No	No	Yes	Yes	Yes	No	No
VA/DoD 2010 [53]	Yes	No	No	No	No	Yes, but as risk factor for suicide in patients with bipolar disorder	No	No
Singapore MOH 2011 [54]	Yes	No	No	No	No	No	No	No
NICE 2014 [55]	Yes	No	No	No	No	Yes	Yes	No
BAP 2016 [56]	Yes	No	No	No	No	No	No	No
CANMAT and ISBD 2018 [57]	Yes	No	No	No	No	No	No	No
BAP 2012 [40]	No	No	No	No	No	No	No	No
WFSBP 2017 [41]	Yes	No	No	No	No	No	No	No
gov.uk 2017 [42]	Yes	Yes	Yes	Yes	Yes	Yes	Yes	Yes
DGPPN and DG-Sucht 2017 [43]	No	No	No	No	No	No	No	No
NICE 2011 [44]	Yes	Yes	No	Yes	Yes	Yes	Yes	Yes
APA 2018 [45]	Yes	No	No	No	No	No	No	No
ASAM 2015 [46]	Yes	Yes	No	No	No	No	No	No

*AOD* Alcohol and other drug, *APA* American Psychiatric Association, *ASAM* American society of addiction medicine, *AUD* alcohol use disorder, *BAP* British Association of psychopharmacology, *CANMAT and ISBD* Canadian Network for Mood and Anxiety Treatments and International Society for Bipolar Disorders, *gov.UK* United Kingdom guidelines on clinical management, *DGPPN and DG-Sucht* German Association for Psychiatry, Psychotherapy, and Psychosomatics and the German Association for Addiction Research and Therapy, *NICE* National Institute for Health and Care Excellence, *RANZCP* Royal Australian and New Zealand College of Psychiatrists, *SIGN* Scottish Intercollegiate Guidelines Network, *Singapore MOH* Singapore Ministry of Health, *SMI* Severe mental illness, *SUD* Substance use disorder, *UK* United Kingdom, *US* United States, *VA/DoD* Department of Veterans Affairs and The Department of Defense, *WFSBP* World Federation of Societies of Biological Psychiatry

All of the guidelines pertaining to coexisting disorders, four (36%) of the SMI guidelines [47, 49, 50, 52], and two (29%) of the SUD guidelines [42, 44] attempted to inform the healthcare providers about the risk of homelessness as being a negative social outcome for individuals affected by SMI or SUD. However, only the Australian government mentioned the risk of homelessness in patients with coexisting disorders, but did not provide further recommendations about how such patients could receive support [39] (Table 7). Assessment of the history of any kind of abuse suffered by the patient, including sexual abuse were only rarely considered [37, 39, 42, 44, 52, 53, 55] (Table 7).

Issue of stigma and discrimination from healthcare providers were covered well by guidelines for co-existing disorders but less so by either SMI or SUD guidelines (Table 7).

Two (67%) of the guidelines pertaining to coexisting disorders, two (18%) of the SMI guidelines, and two (29%) of the SUD guidelines seemed to encourage seeking support from voluntary organizations [37, 38, 42, 44, 48, 50] (Table 7).

## Discussion

This study provides an up-to-date assessment of the scope, quality and inclusivity of international clinical guidelines on mental health and/or substance abuse in relation to diagnosis and treatment of such co-existing disorders and consideration of wider social and contextual issues in treatment recommendations.

The overall quality of the included guidelines rated from a high to moderate quality. The ‘scope and purpose’ and ‘clarity of presentation’ domains were well addressed by the included guidelines. Previous systematic reviews have also demonstrated that clinical guidelines often score high in these domains [58–60]. For the ‘Stakeholder involvement’, it was noticed that there was a lack of incorporation of patient or public preferences in the guidelines development process. The ‘applicability’ domain was rated low amongst all the guidelines.

This review has demonstrated that there is a lack of clinical guidelines aimed to help healthcare professionals manage the dual diagnosis. More importantly any existing single disorder guidelines should incorporate coexisting disorders in diagnosis and treatment recommendations. These guidelines need to be consistent with current evidence that supported development of integral treatment model, strengthen the connection between mental health care setting and substance abuse services, and providing care for patients’ multiple disadvantages including wider social and contextual factors such as homelessness, involvement with criminal justice system [2, 15, 17].

### Implication of practice and research

Until recently, most of the guidelines and recommendations addressed a single disorder; namely, either SMI or SUD. The result of this review suggests that a greater number of guidelines are required in order to cover dual diagnosis given the high overlap of the concurrent disorders.

Most single disorder guidelines included in this review did emphasize the importance of assessment of dual diagnosis. However, treatment adjustment for dual diagnosis was rarely described. Barriers of access to medicines, adherence issues requiring long acting depot injections, and drug interactions (including interactions with drug and substance of abuse) are key issues that require further considerations in single disorder guidelines.

There needs to be better emphasis on the integrated and inclusive care to be offered to the patients with dual diagnosis. Evidence suggests significant reductions in substance abuse, improvement in psychiatric symptoms, quality of life as well as social outcomes in relation to integrated models of management [61, 62]. However, traditional culture of specialist treatment centres that are focused on the treatment of a single condition, lack of expertise and resources are some of the barriers to provision of integrated care as described in the literature [29]. This review suggests that lack of clinical guidelines to offer integrated care could be contributing to the fragmented care. The need for liaison with emergency department, primary care, drug and alcohol services and hospital and specialist treatment centers also require further emphases. There is also scope to enhance cultural and ethnic specific issues in treatment recommendations.

It is well documented in the evidence that the treatment of coexisting disorders multifaceted and requires the continued assessment of many social and contextual issues of a patient. Social and contextual factors were not however uniformly addressed in the included guidelines. While risk of homelessness in patients with SMI, SUD or dual diagnosis was commonly described, further information to health providers to support prevention actions were often missing. It is imperative to signpost patients to housing assistance, volunteer sectors and social benefits system in order to prevent homelessness including repeat cycle of homelessness. Adequate evidence exist on the overlap between homelessness, SUD, SMI and dual diagnosis [63]. Persons who are homeless or risk facing homelessness often find accessing services difficult and future guidelines should consider addressing access issues better [21–23]. These include perceived stigma and discrimination in healthcare setting. Some guidelines described risks of homelessness with dual diagnosis. There are various barriers which patients experiencing homelessness and SUD must overcome in order to obtain housing due to their criminal record and economic status, all of which make them more susceptible to being submerged in their current negative environment and seem to increase the risk of relapse [64, 65].

Only a limited number of guidelines considered the continuity of care of offenders in community settings. It is known that treatment failure can trigger a return back to the patient’s offending behavior after their release from prison [66, 67].

There needs to be better emphases on the integrated and inclusive care to be offered to the patients with dual diagnosis. Liaison with emergency department, primary care, drug and alcohol services and hospital and specialist treatment centers require further emphases. There is also scope to enhance cultural and ethnic specific issues in treatment recommendations. Roles of community based services such as community pharmacy and voluntary sectors should be better stipulated in the guidelines [68–71].

Future research is need needed to cover healthcare professional, patient, carer and payer’s perspectives to identify ways to strengthen the guidelines and limitations and improve patient experiences of care and outcomes. It is also imperative to compare practices against the guideline recommendations. For example, research suggest that patients prescribed antipsychotic medicines are often poorly followed up for their cardiovascular and metabolic health in contrary to the recommendations from the guidelines [72]. Guideline development procedures should learn and share best practices being adopted in other countries.

The assessment of the quality of the guidelines using Agree II checklist suggested that the ‘Rigour of development’ domain scores were generally low as 15 out of 21 included guidelines rated below 70%. This domain captures how well did the guidelines provide evidence in relation to systematic search of relevant body of evidence-based literature, critical appraisal and expert review of the evidence. Further systematic and transparent approach needs to be adopted around the use and reporting of how evidence informed the guideline development.

In summary, this study reinforces the need for adaptation of international clinical guidelines so that healthcare professionals in diverse settings can undertake comprehensive assessment of patient with either SMI or SUD for dual diagnosis, consider assessment of wider social circumstances and consequences that are relevant to the dual diagnosis and adapt their treatment plans accordingly allowing better outcomes for patients, mitigate relapse of SMI, prevent repeat cycles of substance abuse and social consequences such as homelessness. This in turn have the potential to minimize healthcare costs and resource implications. Stakeholder should be involved in development of guidelines.

### Study strengths and limitations

This is the first systematic review to discuss coexisting disorders and aspects of their different complex needs. A comprehensive search was undertaken using databases and professional body web pages. Validated appraisal tool (AGREE II) was used for quality assessment. However, our search was restricted to English language guidelines only. In addition, we did not assess any supplementary patient screening, risk assessment and patient placement criteria that were not included or appended within the published guidelines.

## Conclusion

Treatment guidelines for management of either SUD or SMI have tend to have limited considerations for dual diagnosis. There is a need for the guidelines to be more inclusive in order to enable better diagnosis and treatment and cover social cause and consequences of dual diagnosis such as homelessness. Further emphasis is also needed to promote effective transition of care across services and promotion of self-care after discharge. Professional societies should better communicate the guideline development process as well as rigour in relation to the inclusion and appraisal of evidence base in the guideline development process.

## Supplementary Information

**Additional file 1.** Electronic supplemental material 1: PRISMA Checklist.

**Additional file 2.** Electronic supplemental material 2: Search strategy.

**Additional file 3.** Electronic supplemental material 3: AGREE II Score Sheet.

## Data Availability

All data generated or analyzed during this study are included in this published article [and its supplementary information files].

## Abbreviations

SMI: Severe mental illness
SUD: Substance use disorder
NICE: National Institute for Health and Care Excellence
APA: American Psychiatric Association
US: United States
UK: United Kingdom
GPs: General practitioners
DSM-5: Diagnostic and Statistical Manual of Mental Disorders-5
BAP: British Association of psychopharmacology
AUD: Alcohol use disorder
PRISMA: Preferred Reporting Items for Systematic Reviews and Meta-Analyses
PROSPERO: International Prospective Register of Systematic Reviews
SIGN: Scottish Intercollegiate Guidelines Network
AGREE II: Appraisal of Guidelines for Research & Evaluation II
AOD: Alcohol and other drug
WFSBP: World Federation of Societies of Biological Psychiatry
DGPPN and DG-Sucht: German Association for Psychiatry, Psychotherapy, and Psychosomatics and the German Association for Addiction Research and Therapy
ASAM: American society of addiction medicine
Singapore MOH: Singapore Ministry of Health
VA/DoD: Department of Veterans Affairs and The Department of Defense
CANMAT and ISBD: Canadian Network for Mood and Anxiety Treatments and International Society for Bipolar Disorders
RANZCP: Royal Australian and New Zealand College of Psychiatrists
Gov.uk: United Kingdom guidelines on clinical management
CAMHS: Child and adolescent mental health services
