# Supplementary material for: Scope, quality and inclusivity of international clinical guidelines on mental health and substance abuse in relation to dual diagnosis, social and community outcomes: a systematic review
Source: BMC Psychiatry. 2021 Apr 23;21:209. doi: 10.1186/s12888-021-03188-0 (PMC8066498; doi:10.1186/s12888-021-03188-0)
Supplement: Supplementary file 1 — Additional file 1. Electronic supplemental material 1: PRISMA Checklist. [file 12888_2021_3188_MOESM1_ESM.docx]

**Electronic supplementary material 1: Literature search strategy**

| Database | Search strategy |
| --- | --- |
| **Ovid MEDLINE(R)** | 1 exp *"Bipolar and Related Disorders"/, 2 exp *Bipolar Disorder/, 3 bipolar.ab,ti.,4 bipolar disorder.ab,ti., 5 psychosis.ab,ti., 6 exp *Psychotic Disorders/, 7 exp *Affective Disorders Psychotic/, 8 psychotic.ab,ti., 9 psychotic disorder.ab,ti., 10 exp *Schizophrenia/, 11 exp *Schizophrenia Disorganized/, 12 exp *Schizophrenia, Paranoid/, 13 exp *Schizophrenia, Catatonic/, 14 Schizophrenia.ab,ti., 15 severe mental illness.ab,ti., 16 1 or 2 or 3 or 4 or 5 or 6 or 7 or 8 or 9 or 10 or 11 or 12 or 13 or 14 or 15, 17 addiction.ab,ti., 18 exp *Alcoholism/, 19 alcohol abuse.ab,ti., 20 exp *Substance-Related Disorders/, 21 exp *Alcohol Drinking/, 22 alcohol addict.ab,ti., 23 alcohol dependence.ab,ti., 24 exp *Alcohol-Related Disorders/, 25 alcohol misuse.ab,ti., 26 alcohol related disorder.ab,ti., 27 alcohol disorder.ab,ti., 28 alcoholic.ab,ti., 29 exp *Alcoholics/, 30 alcoholism.ab,ti., 31 exp *Binge Drinking/, 32 exp *Alcoholic Intoxication/, 33 binge drinking.ab,ti., 34 exp *Prescription Drug Diversion/, 35 controlled drug diversion.ab,ti., 36 drug abuse.ab,ti., 37 drug addict.ab,ti., 38 drug dependence.ab,ti., 39 exp *Drug Misuse/, 40 exp *Prescription Drug Misuse/, 41  exp *Substance Abuse, Intravenous/, 42 drug misuse.ab,ti., 43 exp *Illicit Drugs/, 44 drug of abuse.ab,ti., 45 drugs of dependence.ab,ti., 46 hazardous drinking.ab,ti., 47 Illegal drug.ab,ti., 48 exp *Alcohol-Related Disorders/, 49 Illicit drug.ab,ti., 50 exp *Opioid-Related Disorders/, 51 opioid dependence.ab,ti., 52 exp *Substance Withdrawal Syndrome/, 53  opioid withdrawal.ab,ti., 54 drug overdose.ab,ti., 55 exp *Drug Overdose/, 56 drug overuse.ab,ti., 57 polysubstance.ab,ti., 58 Prescription drug abuse.ab,ti., 59 prescription drug diversion.ab,ti., 60 problem substance.ab,ti., 61 severe opioid intoxication.ab,ti., 62 substance abuse.ab,ti., 63 substance abuse, intravenous.ab,ti., 64 exp *Substance Abuse, Oral/, 65 substance abuse, oral.ab,ti., 66 substance addict.ab,ti., 67 substance dependence.ab,ti., 68 exp *Psychoses, Substance-Induced/, 69 Substance induced.ab,ti., 70 substance related disorder.ab,ti., 71 17 or 18 or 19 or 20 or 21 or 22 or 23 or 24 or 25 or 26 or 27 or 28 or 29 or 30 or 31 or 32 or 33 or 34 or 35 or 36 or 37 or 38 or 39 or 40 or 41 or 42 or 43 or 44 or 45 or 46 or 47 or 48 or 49 or 50 or 51 or 52 or 53 or 54 or 55 or 56 or 57 or 58 or 59 or 60 or 61 or 62 or 63 or 64 or 65 or 66 or 67 or 68 or 69 or 70, 72 16 and 71, 73 limit 72 to (english language and yr="2010 -Current") |
| **APA PsychInfo + EMBASE** | 1 bipolar disorder.ab,ti., 2 bipolar.ab,ti.3 psychosis.ab,ti., 4 (bipolar and related disorder).ab,ti., 5 psychotic disorder.ab,ti., 6 psychotic affective disorder.ab,ti., 7 psychotic.ab,ti., 8 Schizophrenia.ab,ti., 9 disorganized Schizophrenia.ab,ti., 10 paranoid Schizophrenia.ab,ti., 11 catatonic Schizophrenia.ab,ti., 12 severe mental illness.ab,ti., 13 1 or 2 or 3 or 4 or 5 or 6 or 7 or 8 or 9 or 10 or 11 or 12, 14 addiction.ab,ti., 15 alcoholism.ab,ti., 16 alcohol abuse.ab,ti., 17 substance related disorder.ab,ti., 18 alcohol drinking.ab,ti., 19 alcohol addict.ab,ti., 20 alcohol dependence.ab,ti., 21 alcohol related disorder.ab,ti., 22 alcohol misuse.ab,ti., 23 alcohol disorder.ab,ti., 24 alcoholic.ab,ti., 25 binge drinking.ab,ti., 26 alcoholic intoxication.ab,ti., 27 prescription drug diversion.ab,ti., 28 controlled drug diversion.ab,ti., 29 drug abuse.ab,ti., 30 drug addict.ab,ti., 31 drug dependence.ab,ti., 32 drug misuse.ab,ti., 33 prescription drug misuse.ab,ti., 34 substance abuse, intravenous.ab,ti., 35 Illicit drug.ab,ti., 36 Illegal drug.ab,ti., 37 drug of abuse.ab,ti., 38 drug of dependence.ab,ti., 39 hazardous drinking.ab,ti., 40 opioid -related disorders.ab,ti., 41 opioid dependence.ab,ti., 42 substance withdrawal syndrome.ab,ti., 43 opioid withdrawal.ab,ti., 44 drug overdose.ab,ti., 45 drug overuse.ab,ti., 46 polysubstance.ab,ti., 47 Prescription drug abuse.ab,ti., 48 problem substance.ab,ti., 49 severe opioid intoxication.ab,ti., 50 substance abuse.ab,ti., 51 substance abuse, oral.ab,ti., 52 substance addict.ab,ti., 53 substance dependence.ab,ti., 54 substance induced psychosis.ab,ti., 55 substance induced.ab,ti., 56 substance related disorder.ab,ti., 57 14 or 15 or 16 or 17 or 18 or 19 or 20 or 21 or 22 or 23 or 24 or 25 or 26 or 27 or 29 or 30 or 31 or 32 or 33 or 34 or 35 or 36 or 37 or 38 or 39 or 40 or 41 or 42 or 43 or 44 or 45 or 46 or 47 or 48 or 49 or 50 or 51 or 52 or 53 or 54 or 55 or 56, 58 13 and 57, 59 limit 58 to english language, 60 limit 59 to yr="2010 -Current", 61 screening.ab,ti., 62 diagnosis.ab,ti., 63 dual diagnosis.ab,ti., 64 disease management.ab,ti., 65 medication therapy management.ab,ti., 66 medical practice management.ab,ti., 67 management.ab,ti., 68 therapeutics.ab,ti., 69 treatment.ab,ti., 70 referral.ab,ti., 71 61 or 62 or 63 or 64 or 65 or 66 or 67 or 68 or 69 or 70, 72 13 and 57 and 71, 73 limit 72 to english language, 74 limit 73 to yr="2010 -Current" |
