# Supplementary material for: Scope, quality and inclusivity of international clinical guidelines on mental health and substance abuse in relation to dual diagnosis, social and community outcomes: a systematic review
Source: BMC Psychiatry. 2021 Apr 23;21:209. doi: 10.1186/s12888-021-03188-0 (PMC8066498; doi:10.1186/s12888-021-03188-0)
Supplement: Supplementary file 3 — Additional file 3. Electronic supplemental material 3: AGREE II Score Sheet. [file 12888_2021_3188_MOESM3_ESM.docx]

**Electronic supplementary material 3: AGREE II score sheet**

| **Domain** | **Item** | **AGREE II Rating** | | | | | | | | | |
| --- | --- | --- | --- | --- | --- | --- | --- | --- | --- | --- | --- |
|  |  | **1** *Strongly Disagree* | | **2** | **3** | **4** | | **5** | **6** | **7** *Strongly Agree* | |
| Scope and purpose | 1. The overall objective(s) of the guideline is (are) specifically described. |  | |  |  | |  |  |  |  | |
|  | 1. The health question(s) covered by the guideline is (are) specifically described. |  | |  |  | |  |  |  |  | |
|  | 1. The population (patients, public, etc.) to whom the guideline is meant to apply is specifically described. |  | |  |  | |  |  |  |  | |
| Stakeholder involvement | 1. The guideline development group includes individuals from all the relevant professional groups. |  | |  |  | |  |  |  |  | |
|  | 1. The views and preferences of the target population (patients, public, etc.) have been sought. |  | |  |  | |  |  |  |  | |
|  | 1. The target users of the guideline are clearly defined. |  | |  |  | |  |  |  |  | |
| Rigor of development | 1. Systematic methods were used to search for evidence. |  | |  |  | |  |  |  |  | |
|  | 1. The criteria for selecting the evidence are clearly described. |  | |  |  | |  |  |  |  | |
|  | 1. The strengths and limitations of the body of evidence are clearly described. |  | |  |  | |  |  |  |  | |
|  | 1. The methods for formulating the recommendations are clearly described. |  | |  |  | |  |  |  |  | |
|  | 1. The health benefits, side effects and risks have been considered in formulating the recommendations. |  | |  |  | |  |  |  |  | |
|  | 1. There is an explicit link between the recommendations and the supporting evidence. |  | |  |  | |  |  |  |  | |
|  | 1. The guideline has been externally reviewed by experts prior to its publication. |  | |  |  | |  |  |  |  | |
|  | 1. A procedure for updating the guideline is provided. |  | |  |  | |  |  |  |  | |
| Clarity of presentation | 1. The recommendations are specific and unambiguous. |  | |  |  | |  |  |  |  | |
|  | 1. The different options for management of the condition or health issue are clearly presented. |  | |  |  | |  |  |  |  | |
|  | 1. Key recommendations are easily identifiable. |  | |  |  | |  |  |  |  | |
| Applicability | 1. The guideline describes facilitators and barriers to its application. |  | |  |  | |  |  |  |  | |
|  | 1. The guideline provides advice and/or tools on how the recommendations can be put into practice. |  | |  |  | |  |  |  |  | |
|  | 1. The potential resource implications of applying the recommendations have been considered. |  | |  |  | |  |  |  |  | |
|  | 1. The guideline presents monitoring and/ or auditing criteria. |  | |  |  | |  |  |  |  | |
| Editorial independence | 1. The views of the funding body have not influenced the content of the guideline. |  | |  |  | |  |  |  |  | |
|  | 1. Competing interests of guideline development group members have been recorded and addressed. |  | |  |  | |  |  |  |  | |
| Overall Guideline Assessment | 1. Rate the overall quality of this guideline. | **1**  *Lowest possible quality* | | **2** | **3** | | **4** | **5** | **6** | **7** *Highest possible quality* | |
| Overall Guideline Assessment | 1. I would recommend this guideline for use. | *Yes* | *Yes, with modifications* | | | | | | | | *No* |
